# Supplementary material for: Mathematical Modeling of Cortical Neurogenesis Reveals that the Founder Population does not Necessarily Scale with Neurogenic Output
Source: Cereb Cortex. 2018 Apr 21;28(7):2540–50. doi: 10.1093/cercor/bhy068 (PMC5998983; doi:10.1093/cercor/bhy068)
Supplement: Supplementary Data [file bhy068suppl_1.zip › Picco_CerebralCortex_SupplMat.pdf]

Mathematical modelling of cortical neurogenesis reveals that the founder population does not necessarily scale with neurogenic output.

Noemi Picco, Fernando García-Moreno, Philip K. Maini, Thomas E. Woolley and Zoltán Molnár

Supplemental Information

Time dependent probability functions

$$\alpha(t) = \begin{cases} \alpha_0 + \frac{\alpha_S - \alpha_0}{t_S - t_0}(t - t_0), & t \in (t_0, t_S), \\ \alpha_S\left(1 - \frac{t - t_S}{t_F - t_S}\right), & t \in (t_S, t_F), \end{cases} \tag{S1}$$

$$\beta(t) = \begin{cases} 0, & t \in (t_0, t_S), \\ \beta_F\left(\frac{t - t_S}{t_F - t_S}\right), & t \in (t_S, t_F), \end{cases} \tag{S2}$$

Local Sensitivity Analysis.

We define sensitivity of the model outcome y to parameter θ as:

$$S = \left|\frac{(y - y^*)/y^*}{(\theta - \theta^*)/\theta^*}\right|, \tag{S3}$$

with $y = \widetilde{N}(t_F) - \frac{\widetilde{N}(t_M)}{\varphi}$ and θ^* indicates the reference value for the parameter whose sensitivity is being calculated. Specifically, θ^* is the parameter value of the optimal strategy (Eq. 5), and y^* the corresponding model outcome.

Full Parameter Search.

The following quantization was used to span the 4-dimensional parameter space:

$$\alpha_0 = 0{:}\,0.1{:}\,1, \quad \alpha_S = \alpha_0{:}\,0.1{:}\,1, \quad \beta_F = 0{:}\,0.1{:}\,1, \quad t_S = t_0{:}\,0.5{:}\,t_F. \qquad \text{(S4)}$$

The notation $(x = x_1{:}\,\Delta x{:}\,x_2)$ indicates that parameter x takes values between $x_1$and $x_2$with incremental steps Δx. Note that any 4-tuple respects equation (2).

The radius of the spheres in Figure S2 is proportional to the relative error:

$$r = \left| \frac{\tilde{N}(t_F) - \frac{\tilde{N}(t_M)}{\varphi}}{\tilde{N}(t_F)} \right|. \qquad \text{(S5)}$$

Note that the numerator is the error as defined in equation (4). Only spheres within 0.1% of relative error are shown, i.e. $r \leq 0.001$.

Analytical Formulation of the Age-dependent Cell Cycle Model.

Species-specific age-dependent cell cycle models are obtained by interpolation of data for mouse and macaque in Kornack and Rakic (1998). Unless otherwise stated, the human age-dependent cell cycle is assumed to follow the same qualitative and quantitative behavior, although stretched over a longer time of neurogenesis (Table 1):

Mouse: $T_C(t) = 10.2 + 2.05 \times (t - 12) \quad t \in (E11,\ E19)$ (S6)

Macaque: $T_C(t) = \begin{cases} 23 + 1.55 \times (t - 40) & t \in (E40, E60) \\ 54 - 1.35 \times (t - 60) & t \in (E60, E95) \end{cases}$ (S7)

Human: $T_C(t) = \begin{cases} 23 + 0.82 \times (t - 40) & t \in (E40, E77.8) \\ 54 - 0.7147 \times (t - 77.8) & t \in (E77.8, E125). \end{cases}$ (S8)

Parameter Estimation with Approximate Bayesian Computation (ABC).

Let y be the data point and $\mathcal{M} = \mathcal{M}(\boldsymbol{\theta})$ a model that we chose to explain the observed data, where $\boldsymbol{\theta} = (\alpha_0, \alpha_S, \beta_F, t_S)$ is the vector of model parameters, taking values in $\Omega \subset \mathbb{R}^4$. We want to estimate values of parameters $\boldsymbol{\theta}$ that best explain the data y, with the given model $\mathcal{M}$. The Approximate Bayesian Computation (ABC) rejection algorithm allows us to build a discrete approximation of the posterior distribution of $\boldsymbol{\theta}$ (Picco et al. 2017). Specifically, the ABC algorithm iteratively draws a proposed 4-tuple of values for $\boldsymbol{\theta}$ from a uniform prior on some region $\overline{\Omega} \subset \Omega$ where conditions in equations (2) are satisfied. If the model, simulated with the proposed parameter set, falls close to the data within tolerance ϵ, then the 4-tuple is accepted, otherwise rejected. The collection of all accepted 4-tuples constitutes an approximation of the posterior distribution. The tolerance ϵ is iteratively adjusted to obtain a target acceptance ratio $\bar{\pi} = 0.0002$, where $\bar{\pi}$ is defined as the number of accepted 4-tuples over the number of proposed 4-tuples.

In this instance, our data point corresponds to the number of neurons at the end of neurogenesis. This value is calculated from the number of neurons in the adult species of interest ($\overline{N}$), adjusted for post-neurogenesis cell death (δ) and neuronal migration (μ):

$$y = N(t_F) = \overline{N} \times (1 + \delta) \times (1 - \mu). \quad \text{(S9)}$$

Unless otherwise stated, we set $\delta = 0.3$ and $\mu = 0.25$. Values for $\overline{N}$ can be found in Table 1.

Captions to Supplementary Figures

Figure S1. Neurogenesis model with constant probabilities α and β. Examples of dynamics obtained with $t_0 = E11, t_F = E19, T_C = 14.3\ hr$ and probabilities α and β in representative regions of the parameter space.

Figure S2. Full search of the parameter space (α_0, α_S, β_F, t_S) to identify the mouse strategy for the constant cell cycle model. The radius r of each sphere is proportional to the error ε (see supplemental information). Only spheres within 0.03% of relative error are shown. The arrow indicates the predicted strategy (i.e. minimum r), corresponding to equation (5). Trivial strategies (TS) are disregarded because they are not biologically relevant. TS1 ($\alpha_0 = \alpha_S = 0, t_S = t_F$) and TS2 ($\alpha_0 = \alpha_S = 0, \beta_F = 0$): all divisions are SymP, no neurons are produced. TS3 ($\beta_F = 0$): progenitors never undergo SymN divisions.

Figure S3. Founder population estimates for mouse, macaque, and human using the corresponding strategy for constant (o) and age-dependent (✫) cell cycle length models. Human estimates are repeated for post-neurogenesis cell death ranging from 30% to 70%. Arrows indicate direction of increasing cell death. **(A)** 25% interneuronal migration. **(B)** 50% interneuronal migration.

Figure S4. Founder population estimates for mouse, macaque, and human using the corresponding strategy for varied human progenitors cell cycle length. Estimates for age-dependent **(A)** and constant **(B)** cell cycle length models. The human cell cycle duration is increased up to twice the macaque value (see colormap).
