# Supplementary figures and images for: Mathematical Modeling of Cortical Neurogenesis Reveals that the Founder Population does not Necessarily Scale with Neurogenic Output
Source: Cereb Cortex. 2018 Apr 21;28(7):2540–50. doi: 10.1093/cercor/bhy068 (PMC5998983; doi:10.1093/cercor/bhy068)

**Figure S1**

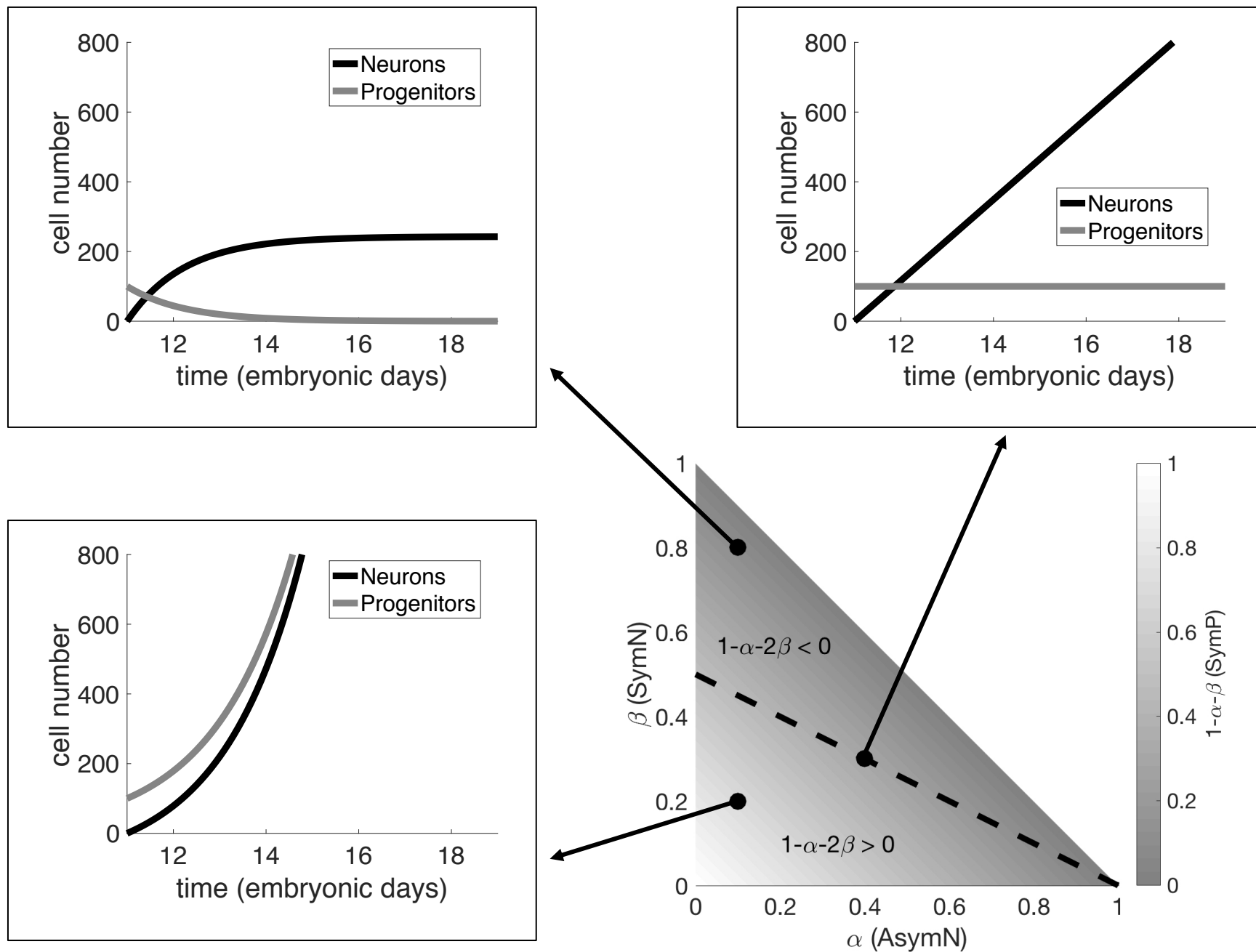

Figure S2

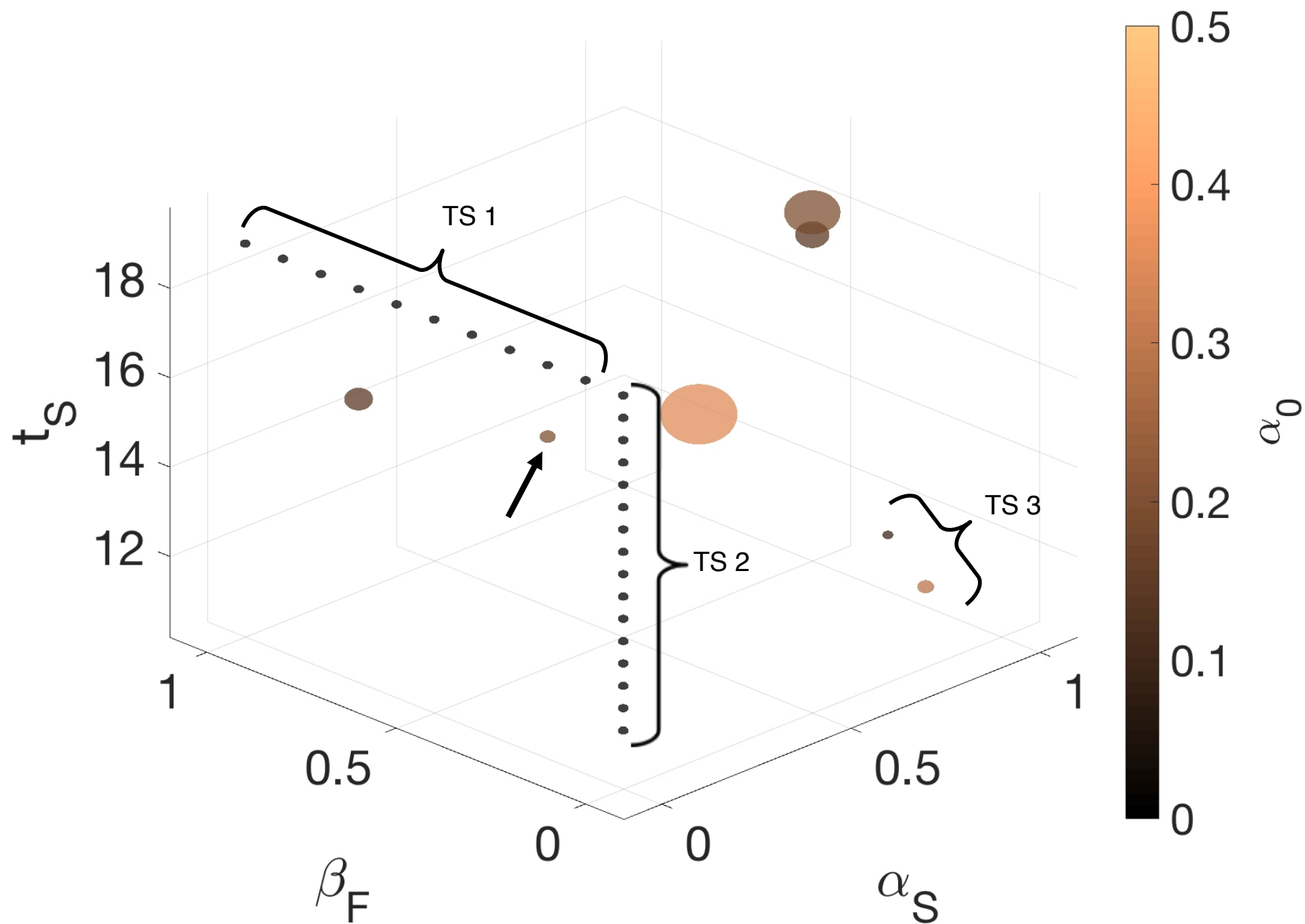

**Figure S3**

**A**

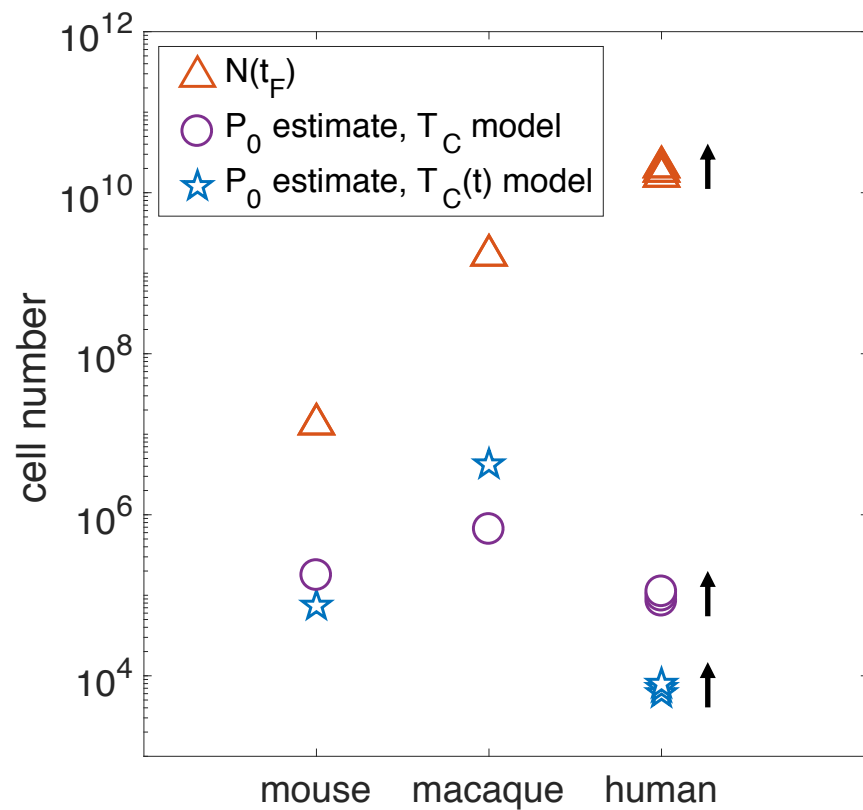

**B**

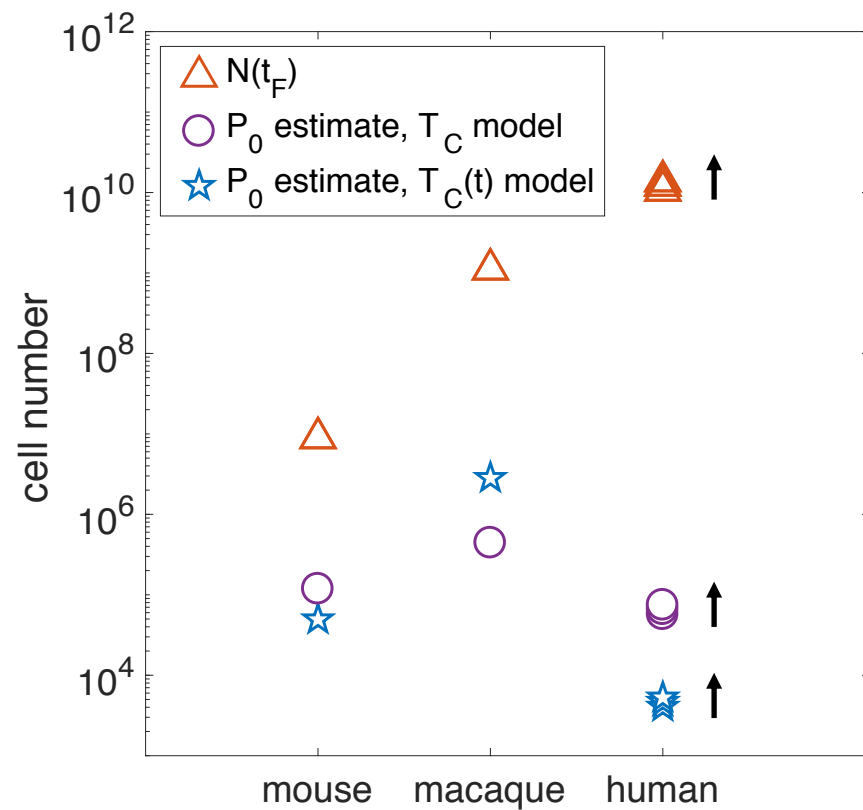

**Figure S4**

**A**

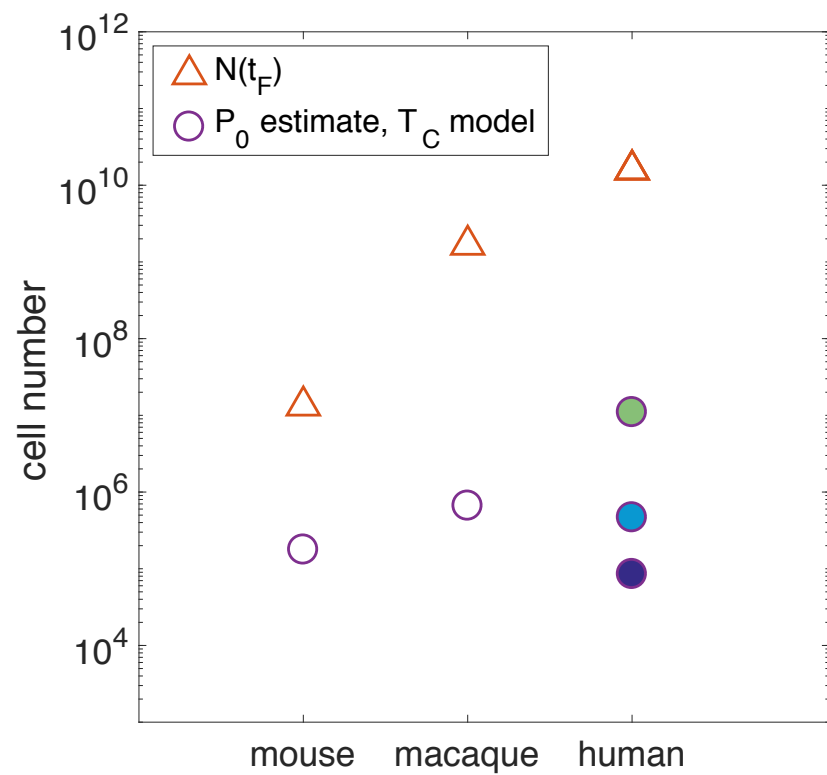

**B**

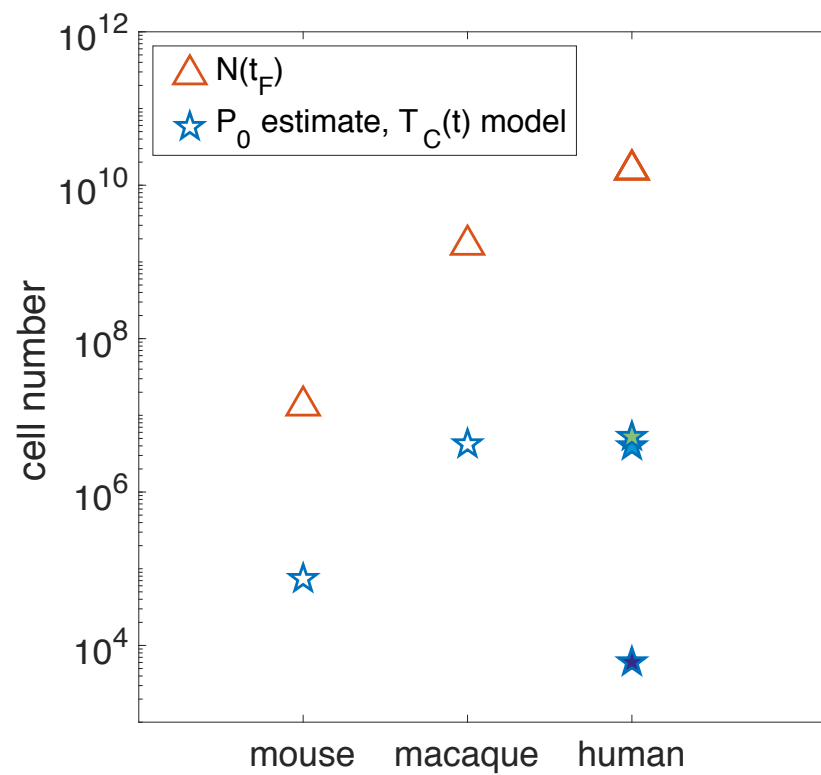

Cell cycle amplification

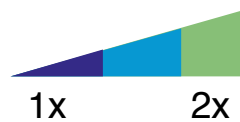

Supplement: Supplementary Data [file bhy068suppl_1.zip › SupFigures_highres.pdf]
